# Clinical practice guidelines: towards better quality guidelines and increased international collaboration

**DOI:** 10.1038/sj.bjc.6601077

**Published:** 2003-08-15

**Authors:** R Grol, F A Cluzeau, J S Burgers

**Affiliations:** 1 University Medical Centre Nijmegen, Nijmegen, The Netherlands; 2 St George's Hospital Medical School, London, UK

**Keywords:** practice guidelines, quality assessment, international network

Over recent decades, the number of available clinical practice guidelines has grown enormously. These guidelines are increasingly used in health-care systems throughout the world to improve the quality of patient care, and this also applies to cancer care. Evidence-based guidelines are seen by professionals, authorities, managers and policy makers as powerful tools for achieving effective and efficient care (Woolf *et al*, 1999). They are considered to be the ideal mediator for bridging the gap between the growing stream of research findings and actual clinical practice. Guidelines should meet specific quality criteria to ensure good quality. Users should be confident that potential biases inherent in guideline development have been addressed appropriately and that the recommendations for practice are both internally and externally valid, as well as feasible for practice (AGREE (Appraisal of Guidelines Research and Evaluation) Collaborative Group, 2000). However, recent studies have reported that the methodological quality of many guidelines is modest and is heterogeneous between the different guidelines and different guideline programmes (Shaneyfelt *et al*, 1999; Grilli *et al*, 2000; Lacasse *et al*, 2001; Burgers *et al*, 2003a). Although clinical guidelines can provide a solution to some of the important problems in patient care, there are issues that need to be tackled before guidelines can achieve their full potential (Grol, 2001a). We will start by outlining these problems, and then we will present a set of criteria for high-quality guidelines developed and validated by an international group of researchers and guideline developers (the AGREE collaboration). Some cancer guidelines (including those produced by the French National Federation of Cancer Centres–FNCLCC the SOR) were used in the validation process for these criteria. We will then provide some recommendations for guideline developers with the aim that this will help researchers and practitioners in cancer care to develop high-quality guidelines for the management of their patients.

## PROBLEMS WITH GUIDELINES

Various problems with guidelines and their development that can impede their maximal use and profit have been reported:

*Lack of quality*: There are currently too many low-quality guidelines. There seems to be a ‘guideline industry’ emerging in many Western countries with a considerable variation in guidelines from different sources (Grol *et al*, 1998a). Physicians and other professionals are probably overwhelmed by all these guidelines, particularly since guidelines on the same topic sometimes present different recommendations for practice (Fahey and Peters, 1996; Psaty and Furberg, 1999). Many of the current guidelines have not been developed in a rigorous and systematic way, and are not based on the best evidence or present the vested interests of specific parties, including health-care industries. A series of recent studies assessing the quality of clinical guidelines show that many guidelines do not meet important quality criteria (Ward and Grieco, 1996; Varonen and Mäkelä 1997; Cluzeau *et al*, 1999; Shaneyfelt *et al*, 1999; Grilli *et al*, 2000; Lacasse *et al*, 2001). The unsystematic development of guidelines can contribute to this low quality (Thomson *et al*, 1998; Burgers *et al*, 2003a).

*Lack of evidence*: A second problem is that despite a rigorous search and analysis of the scientific literature, clear evidence is available for only part of the practical actions and decisions recommended in the guidelines (Vogel *et al*, 2000; Dinkevich *et al*, 2001). There is a large grey area where expert opinions, practitioners' and patients' preferences as well as societal priorities are more important in the development of guidelines than research results (Naylor, 1995, Eccles *et al*, 1998). When evidence is missing, reliable procedures for including expert opinions and stakeholders' preferences are required; such procedures are not present in many guideline development programmes (Burgers *et al*, 2003b).

*Translation of evidence into recommendations for practice*: Even when evidence has been summarised, it is often difficult to translate it into recommendations for practice. Guideline users deal with a more heterogeneous population of patients and more complex health-care processes than those covered in the original research (Van Weel and Knottnerus, 1999; Koes *et al*, 2001). Most cancer clinical research deals with separate diagnostic or treatment decisions in selected samples of patients, while the practice of cancer care usually involves dealing with complex multidisciplinary care processes in a variety of patient groups. There has been very little research into the best way to manage such processes and chains of related actions and decisions by different care providers. Also, it is not easy to translate guideline recommendations into decisions in practice, since guidelines can never cover all the relevant clinical details necessary for individual patients. Most guidelines fail to take these issues into account.

*Interpretation of evidence*: Guidelines are developed by humans and the process is, therefore, prone to errors and subjective interpretations on the one hand and personal values and cultural backgrounds on the other. Even when clear evidence is available, it is often interpreted differently by different guideline developers in different settings from different cultural or professional backgrounds (Fahey and Peters 1996; Koes *et al*, 2001). For example, the USA guidelines for the management of patients with high risk of breast cancer recommend regular self-examination and prophylactic mastectomy (requiring patient consent only). In contrast, the French guidelines do not recommend self-examination (because this may induce fear) and are very strict with regard to prophylactic mastectomy (Eisinger *et al*, 1999). The authors of this study reported that evidence-based guidelines may be a result of specific cultural beliefs.

*Feasibility*: The consequences of guidelines in terms of acceptance by patients, and the resources, staff, skills and equipment needed for implementation are usually not considered during the development process. For example, in a study of a structured method to educate patients with atrium fibrillation about the benefits and risks of anticoagulation treatment, half of the patients did not choose the evidence-based treatment (Howitt and Armstrong, 1999). Another example is the implementation of a dyspepsia guideline in the UK that may have resulted in a three-fold increase in the number of endoscopies (Haycox *et al*, 1999). Whether a society is willing and able to pay the bill for particular innovations cannot be determined on the basis of scientific evidence. Most guidelines do not consider these issues.

*Difficult implementation*: For a long time, most guideline developers assumed that good evidence presented to practitioners in a structured way automatically led to better performance. However, results from many controlled trials and systematic reviews show that efforts to implement guidelines are often not very successful (Bero *et al*, 1998; Wensing *et al*, 1998; Grol and Grimshaw, 1999; Grimshaw *et al*, 2001; Grol 2001b). At best, small to moderate improvements in the care process have been found (usually not more than 5–10%, depending on the implementation methods used), whereas the impact on patient outcomes has often not been studied or proved to be absent (Grimshaw and Russell, 1993; Hunt *et al*, 1998). Issues of implementation are seldom addressed in the development of guidelines.

## CRITERIA FOR GOOD-QUALITY GUIDELINES: THE AGREE INSTRUMENT

To guarantee that clinical practice guidelines can be an effective tool to improve care for (cancer) patients they should meet specific quality criteria (Feder *et al*, 1999; Shekelle *et al*, 1999). This concern is felt worldwide, and has been underlined by renewed calls for internationally recognised standards to promote the rigorous development of clinical guidelines and to assess their quality (Shaneyfelt *et al*, 1999; Grilli *et al*, 2000). Clearly, these standards should be valid, reliable and feasible.

The AGREE Collaboration has recently developed such criteria in the context of an EU-funded research project. Bringing together researchers and policy makers from 12 countries (UK, The Netherlands, Denmark, Finland, France, Switzerland, Spain, Canada, Italy, Germany, USA, New Zealand), the collaboration's aim is to establish comparable frameworks for the assessment and monitoring of the quality of clinical practice guidelines, including the process of development and the reporting of the process. The AGREE Instrument was developed through a multistage process of item generation, selection and scaling, field testing and refinement procedures. A small working group first compiled a comprehensive checklist of 82 items from existing appraisal instruments and relevant literature that covered recognised components of guideline quality. The term ‘quality’ was defined as the confidence that the biases linked to the rigour of development, presentation and applicability of a guideline had been minimised during the development process. Most of the items were derived from existing lists or instruments (e.g. Lohr and Field, 1992; Grol *et al*, 1998b; Cluzeau *et al*, 1999) to cover all aspects of the concept of quality. Following preliminary testing, the checklist was reduced to 32 items classified into five quality domains. This was then circulated to all the members of the AGREE collaboration and other international experts for their comments. The resulting ‘first’ version of the instrument was then field tested for reliability and validity on 100 guidelines with 195 appraisers from 11 countries, with 31 cancer guidelines, including guidelines from the FNCLCC and from the Canadian Cancer Care Ontario Practice Guidelines Initiative. After refinement, the instrument was field tested again on a random sample of 33 guidelines (including 14 cancer guidelines) from the first field test with a new set of appraisers. The results were encouraging and demonstrated that the instrument was easy to use and could be applied consistently to a broad range of guidelines across different countries (AGREE Collaboration, 2003). Generally, the scores for cancer guidelines were high with the instrument (for example, they were higher than the scores for guidelines on diabetes and asthma for rigour of development).

The final AGREE instrument consists of 23 key items (Table 1

**Table 1:** The AGREE instrument

*Scope and purpose*
1. The overall objective(s) of the guideline is (are) specifically described.
2. The clinical question(s) covered by the guideline is (are) specifically described
3. The patients to whom the guideline is meant to apply are specifically described

*Stakeholder involvement*
4. The guideline development group includes individuals from all the relevant professional groups
5. The patients' views and preferences have been sought
6. The target users of the guideline are clearly defined
7. The guideline has been piloted among target users

*Rigour of development*
8. Systematic methods were used to search for evidence
9. The criteria for selecting the evidence are clearly described
10. The methods for formulating the recommendations are clearly described
11. The health benefits, side effects and risks have been considered in formulating the recommendations
12. There is an explicit link between the recommendations and the supporting evidence
13. The guideline has been externally reviewed by experts prior to its publication
14. A procedure for updating the guideline is provided

*Clarity and presentation*
15. The recommendations are specific and unambiguous
16. The different options for management of the condition are clearly presented
17. Key recommendations are easily identifiable
18. The guideline is supported with tools for application

*Applicability*
19. The potential organisational barriers in applying the recommendations have been discussed
20. The potential cost implications of applying the recommendations have been considered
21. The guidelines present key review criteria for monitoring and/or audit purposes

*Editorial independence*
22. The guideline is editorially independent from the funding body
23. Conflicts of interest of guideline development members have been recorded

) categorised into six domains (see: http://www.agreecollaboration. org). Each domain is intended to measure a separate dimension of guideline quality.

*Scope and purpose* (items 1–3): These items are concerned with the overall aim of the guideline, the specific clinical questions and the target patient population.

*Stakeholder involvement* (items 4–7): These items focus on the extent to which the guideline represents the views of its intended users. Guideline development needs to be carried out by a multidisciplinary group involving all stakeholders whose clinical activities are likely to be covered in the proposed guideline. This also includes patient groups.

*Rigour of development* (items 8–14): These items relate to the process used to gather and synthesise the evidence, and the methods used to formulate the recommendations and to update them. The recommendations should be explicitly linked to the supporting evidence. However, because most current guidelines use a mixture of ‘expert’ judgement and literature review, disclosure of disagreement or uncertainties encountered during the development may help to clarify the process. Guidelines should be reviewed externally before publication, and the process used clearly described. They should also always include a date of publication, and because guidelines need to reflect current research, they should contain a clear statement about the updating procedures.

*Clarity and presentation* (items 15–18): These items deal with the language and format of the guidelines. Since the main role of guidelines is to help clinicians and patients make better decisions, busy clinicians need simple, patient-specific, user-friendly guidelines that are easy to understand. Good guidelines present clear information about the management options available and the likely consequences of each. This information can be presented in a variety of formats to suit the needs of the user.

*Applicability* (items 19–21): These items cover the likely organisational, behavioural and cost implications of applying the guidelines. Guidelines should be feasible to use in the current organisation of care and must fit into routine practice and the time constraints present. In addition, review criteria should be developed that link the guideline use to audits and other quality improvement initiatives.

*Editorial independence* (items 22–23): These items assess the independence of the recommendations and acknowledgement of possible conflict of interests for the members of the guideline development group. An increasing number of guidelines are funded directly, or indirectly, by external funding. There should be an explicit statement that the views and/or interests of the funding body have not influenced the final recommendations.

To help users understand the items, the instrument contains a users' guide with explanatory notes. Each item is scored on a reduced four-point Likert scale, and there is an overall rating as to whether the guideline should be recommended or not for use in practice.

The AGREE instrument was developed through a detailed and lengthy process that took many years to complete. Despite this, most of the AGREE quality criteria are still based on theoretical assumptions rather than on empirical evidence. They were developed through discussions between researchers from several countries who have extensive experience and knowledge of clinical guidelines. It remains to be shown that these criteria are actually linked to ‘better’ quality guidelines leading to improved patient care and outcomes. Another issue is that the AGREE instrument relies heavily on the quality of the background documentation on which the guidelines are based. Although defining quality by the rigour of reporting rather than the rigour of content may not provide information on the intrinsic quality of the guidelines, it is clear that without some information about the development process it is impossible to assess the quality of guidelines (Hayward *et al*, 1995). Finally, guidelines need to be used if they are to assist decision-making in practice. Our understanding of what attributes of guidelines determine this complex process is limited, although important research is emerging in the field (Grol *et al*, 1998b; Foy *et al*, 2002). The quality of a guideline is affected by scientific considerations as well as human and practical factors. Future validation research will need to focus on how these elements interact in clinical practice.

## RECOMMENDATIONS FOR GUIDELINE DEVELOPERS

To ensure that guidelines are of high quality, they should be preferably developed within a structured and coordinated guidelines programme (Table 2

**Table 2:** Key criteria for good clinical guideline programmes

*People involved in guideline development*
credible organisation responsible for guideline development
target users involved in guideline development (‘ownership’)
balanced multidisciplinary guideline development group
patient involvement at any stage of the development process

*Methodology of guideline development*
systematic review of literature, including existing high-quality guidelines
combining evidence linkage and expert consensus in formulating recommendations
external peer review
formal update procedure
use of quality criteria for guidelines and guideline development

*Dissemination and implementation strategy*
production of different formats of the guideline, including patient versions, and tools for applications
use of the Internet
multiple implementation strategies
review criteria, indicators for assessing the use of guidelines

). A recent French ‘before–after’ controlled study confirmed the positive impact of the newly established SOR guidelines programme on medical practice for cancer management (Ray-Coquard *et al*, 2002). Sufficient budget and resources are also needed (Shekelle *et al*, 1999). However, substantial savings could be made by active cooperation between national and international guideline organisations. This could include the exchange of existing cancer guidelines and evidence reports, collaboration for literature searches for revision of those guidelines, and organising joint peer review of draft guidelines (Browman, 2000). However, effective and efficient collaboration requires that the methodological principles are common. The development of the AGREE instrument, which involved the participation of leaders from various guideline development organizations, revealed an increased international consensus and willingness to work together (Burgers *et al*, 2003b, AGREE Collaboration, 2003). Recent programmes may benefit from the methodology created by more established programmes. However, it must also be kept in mind that each country has its own norms and values that influence the content and presentation of guidelines. Therefore, the aim should not be to develop international guidelines, but to reach international agreement about the requirements for methodology and reporting of guidelines (De Maeseneer and Derese 1999). The AGREE instrument is an excellent aid for improving the reporting of guidelines. For instance, the Scottish Intercollegiate Guideline Network (SIGN) provides a guide with examples derived from SIGN guidelines, adjacent to each item, on how information can be made available (SIGN50, 2001; http://www.sign.ac.uk). Uniform reporting gives a certain guarantee of quality. Moreover, it simplifies the comparison of guidelines for the same clinical conditions. For the development (or revision) of guidelines, the use of existing high-quality guidelines (for instance, guidelines included in the US National Guideline Clearing house) can save a lot of time and effort (Baker and Feder, 1997; Adams *et al*, 1999; http://www.guideline.gov/index .asp). For example, the literature search and review could be used when similar questions are being examined. Above all, it is useful to see how other guideline development groups have collected and interpreted the evidence and how they have translated the evidence into recommendations. After publication, guidelines must be disseminated and implemented effectively (Grol, 2001b). Guideline developers should be aware of the potential facilitators for and barriers to implementation when they are formulating the recommendations. If substantial changes in practice are necessary to apply the recommendations, additional information should be added with practical suggestions, for example, about improved organisation of the care processes. They should also pay particular attention to the format and presentation of the guideline, for example, by providing short summaries that can be easily used during contacts with patients (Hayward *et al*, 1997; Jackson and Feder, 1998). Furthermore, application tools should be developed, such as indicators for performance assessment, teaching materials, patient information pamphlets, or computer decision-aids. It is important to involve the end users in the development process to ensure local acceptance and relevance to local practice (Browman, 2001).

A final, but important, consideration is the need to keep guidelines up-to-date. Shekelle *et al* (2001a) presented a model for assessing the validity of guidelines based on a combination of multidisciplinary expert opinion and literature searches. The use of recent systematic reviews can considerably limit the workload of literature searching (Cook *et al*, 1997; Silagy *et al*, 2001). Based on a review of new evidence, the update may be major or minor. It has been suggested that, in principle, the update procedure should be performed every three years (Shekelle *et al*, 2001b).

## NEW DEVELOPMENTS

Clinical practice guidelines should meet specific quality criteria if they are to be valuable tools in the care for cancer patients. These criteria have been defined and validated by the AGREE Collaboration (see Table 1). Better collaboration between guideline developers throughout the world is important to avoid unnecessary duplication of effort. One such collaboration, the ‘International Guidelines Network (GIN–http://www.g-i-n.net/)’, is currently being established and will be operational soon. Another collaboration, specifically for cancer guidelines, is also being prepared and will apply for funding under the European Union's 6th Framework Programme. Such networks will provide a platform for international information exchange and collaborative research. These efforts will apply to existing guidelines or guidelines under development, guidelines reviews, methodological information (for example, a guide for guideline developers) and tools for application and evaluation of guidelines. We expect that many guideline organisations throughout the world will join the proposed networks.
